# Effect of grape pomace supplement on growth performance, gastrointestinal microbiota, and methane production in Tan lambs

**DOI:** 10.3389/fmicb.2023.1264840

**Published:** 2023-09-28

**Authors:** Xindong Cheng, Xia Du, Yanping Liang, Abraham Allan Degen, Xiukun Wu, Kaixi Ji, Qiaoxian Gao, Guosheng Xin, Haitao Cong, Guo Yang

**Affiliations:** ^1^Key Laboratory of Stress Physiology and Ecology of Gansu Province, Northwest Institute of Eco-Environment and Resources, Chinese Academy of Sciences, Lanzhou, China; ^2^University of Chinese Academy of Sciences, Beijing, China; ^3^Desert Animal Adaptations and Husbandry, Wyler Department of Dryland Agriculture, Jacob Blaustein Institutes for Desert Research, Ben-Gurion University of the Negev, Beer Sheva, Israel; ^4^Key Laboratory of Extreme Environmental Microbial Resources and Engineering, Lanzhou, China; ^5^Ningxia Feed Engineering Technology Research Center, Ningxia University, Yinchuan, China; ^6^Shandong Huakun Rural Revitalization Institute Co., Ltd., Jinan, China; ^7^Yellow River Estuary Tan Sheep Institute of Industrial Technology, Dongying, China

**Keywords:** wine by-product, rumen, hindgut, microbiome, methanogenesis

## Abstract

Grape pomace (GP), a by-product in wine production, is nutritious and can be used as a feed ingredient for ruminants; however, its role in shaping sheep gastrointestinal tract (GIT) microbiota is unclear. We conducted a controlled trial using a randomized block design with 10 Tan lambs fed a control diet (CD) and 10 Tan lambs fed a pelleted diet containing 8% GP (dry matter basis) for 46 days. Rumen, jejunum, cecum, and colon bacterial and archaeal composition were identified by 16S rRNA gene sequencing. Dry matter intake (DMI) was greater (*p* < 0.05) in the GP than CD group; however, there was no difference in average daily gain (ADG, *p* < 0.05) and feed conversion ratio (FCR, *p* < 0.05) between the two groups. The GP group had a greater abundance of *Prevotella 1* and *Prevotella 7* in the rumen; of *Sharpe*, *Ruminococcaceae 2*, and *[Ruminococcus] gauvreauii group* in the jejunum; of *Ruminococcaceae UCG-014* and *Romboutsia* in the cecum, and *Prevotella UCG-001* in the colon; but lesser *Rikenellaceae RC9 gut group* in the rumen and cecum, and *Ruminococcaceae UCG-005* and *Ruminococcaceae UCG-010* in the colon than the CD group. The pathways of carbohydrate metabolism, such as L-rhamnose degradation in the rumen, starch and glycogen degradation in the jejunum, galactose degradation in the cecum, and mixed acid fermentation and mannan degradation in the colon were up-graded; whereas, the pathways of tricarboxylic acid (TCA) cycle VIII, and pyruvate fermentation to acetone in the rumen and colon were down-graded with GP. The archaeal incomplete reductive TCA cycle was enriched in the rumen, jejunum, and colon; whereas, the methanogenesis from H_2_ and CO_2_, the cofactors of methanogenesis, including coenzyme M, coenzyme B, and factor 420 biosynthesis were decreased in the colon. The study concluded that a diet including GP at 8% DM did not affect ADG or FCR in Tan lambs. However, there were some potential benefits, such as enhancing propionate production by microbiota and pathways in the GIT, promoting B-vitamin production in the rumen, facilitating starch degradation and amino acid biosynthesis in the jejunum, and reducing methanogenesis in the colon.

## 1. Introduction

In ruminant production, feed typically accounts for 60–70% of total production expenditures (Arthur et al., 2004; Karisa et al., 2014). Therefore, saving feed costs by the use of by-products as a feed source for livestock could increase profits substantially. Grapes are cultivated widely to produce wine, the most popular beverage in the world. According to the Food and Agriculture Organization (FAO), there are 75,866 km^2^ of land worldwide devoted to vineyards, with an annual yield of 74 million tons of grapes, of which approximately 75% is used for winemaking (Beres et al., 2017). In the production of grape juice, whole grape bunches are pressed, and grape pomace (GP), including seeds, skins and stems, remains (Yu and Ahmedna, 2013). The GP, approximately 20% of the total mass, is generally discarded, which can cause serious environmental problems when accumulated.

GP is rich in tannins and other polyphenols. Tannins are high molecular weight polyphenolic compounds that possess antimicrobial, anti-parasitic, anti-viral, antioxidant, anti-inflammatory, and immunomodulatory properties (Besharati et al., 2022). In addition, tannins can increase nitrogen utilization in the gut, improve ruminal fermentation, and decrease methane production in ruminants. However, at high intakes, tannins are considered as anti-nutritional since they bind to proteins, carbohydrates, and other substances, consequently reducing fiber digestibility. GP can be fed to ruminants at approximately 10% of the dry matter intake (Chikwanha et al., 2019; Gao et al., 2019). Previous studies reported that GP could affect nutrient utilization (Abarghuei et al., 2010), enhance meat and milk quality and antioxidant capacity (Ianni and Martino, 2020; Molosse et al., 2021), and reduce methane (CH_4_) emission (Moate et al., 2014; Russo et al., 2017). In addition, GP can improve rumen fermentation, increase propionate concentration to boost growth performance, and decrease methane emission (Khiaosa-ard et al., 2015; Foiklang et al., 2016a). However, at high intakes (usually >20% DM), GP can be detrimental to rumen fermentation and reduce the digestibilities of dry matter (DM), neutral detergent fiber (NDF) and acid detergent fiber (ADF), and the production of volatile fatty acid (VFAs) (Besharati and Taghizadeh, 2009; Ishida et al., 2015).

The mammalian digestive tract is colonized by vast numbers of microorganisms, including bacteria, fungi, archaea, protozoa, and viruses, which outnumber the cells in the body by hundreds of times and are considered the ‘second genome’ of the animal (Zhu et al., 2010). The rumen is a unique organ in ruminants, accounting for approximately 75% of the total volume of the sheep’s stomach (Membrive, 2016). The microbes in the rumen can ferment complex cellulose, starch, proteins, and lipids, producing VFAs, which provide up to 70% of the host’s energy needs, and can synthesize microbial proteins and vitamin B complexes (Mizrahi et al., 2021). Microbes in the lower gut can also degrade feed and produce CH_4_. The jejunum is the main site of digestion and absorption of protein and carbohydrates, while microbial fermentation in the hindgut can degrade up to 30% of cellulose and hemicellulose in ruminants (O’Hara et al., 2020). In addition, archaea in the gastrointestinal tract (GIT) produce large amounts of CH_4_. Enteric CH_4_ from ruminants accounts for 73% of livestock emissions (Rodrigues, 2016), and the CH_4_ emission is the equivalent of 2–12% of the digestible energy intake of the host (Johnson and Johnson, 1995). The rumen is the primary site of methane production in sheep, while the hindgut contributes only 6–14% of methane production (Immig, 1996).

The effect of GP on rumen and feces microbiota has been studied (Kafantaris et al., 2017; Biscarini et al., 2018), but the results have been equivocal. GP may potentially enhance the abundance of beneficial bacteria and suppress methane emission (Ross et al., 2013), or have no impact on the composition and function of rumen microbiota (Buffa et al., 2020). There has been no comprehensive study on the potential shaping effects of feed supplemented with GP on the GIT microbiome. To fill this gap, we examined the effect of GP on bacteria and archaea in different segments of the GIT of Tan lambs. We hypothesized that adding GP (8%) would enhance sheep growth, have a positive impact on gastrointestinal microbe fermentation, and reduce methane production.

## 2. Materials and methods

The protocol of the study and all procedures on the lambs complied with animal research and welfare recommendations and were approved by the Academic Committee of the Northwestern Institute of Eco-Environment Resources, Chinese Academy of Sciences (protocol number: CAS201810082).

### 2.1. Experimental design, diets, and management

Twenty castrated, 3-month-old Tan lambs of similar body condition and weight (22 ± 1.7 kg) were divided randomly into two groups (a separate sheepfold for each group) of 10 lambs each: one group was fed a control diet (CD) and one group was fed a diet containing 8% GP by DM. The diets were offered twice daily, at 8:00 and 18:00, with free access to water and lick mineral blocks for 46 d. The GP was bought from a winery at the eastern foot of the Helan Mountain, and after drying, the feed was prepared by the Feed Engineering Technology Research Center of Ningxia University. The two diets were iso-nitrogenous, with the same energy yield and were prepared according to the Feeding Standards for sheep and goats (Ministry of Agriculture of China, 2005; Table 1). The lambs were weighed before morning feed was offered on days 0 and 47 to calculate average daily gain (ADG). All feed offered and feed remains before the feed was offered each morning were weighed to determine daily dry matter intake (DMI). Feed conversion ratio (FCR) was calculated as the ratio between DMI (g/d) and ADG (g/d).

**Table 1:** Ingredients and chemical composition of the control (CD) and grape pomace (GP) diets.

Ingredient (g/kg DM)	CD	GP
Corn	392.6	299.7
Corn straw	370	370
GP^1^	0	80^1^
Cotton meal	74.5	73.0
Soybean meal	53.1	53.1
DDGS	30	30
Alfalfa	30	30
Premixture^2^	20	20
Soybean oil	1.8	16.2
Sunflower meal	10	10
Limestone	9	9
Saleratus	5	5
Salt	3	3
Urea	1	1
Chemical composition (g/kg DM)		
Crude protein	139	139
NDF	327	357
ADF	208	210
Digestible energy (MJ/kg)	9.0	9.0
Calcium, %	6.7	6.9
Phosphorus, %	4.0	3.9

1 The composition of GP (g/kg DM) was 122 crude protein, 306 crude fiber, 575 NDF, 387 ADF, 82.2 crude fat, 4.4 calcium, 3.1 phosphorus, and 6.83 MJ/kg digestible energy.

2 Premixture composition per kg dry matter: vitamin A, 180000 IU; vitamin D_3_, 80,000 IU; vitamin B, 800 IU; Cu (as copper sulfate), 100 mg; Fe (as ferrous sulfate), 300 mg; Zn (as zinc sulfate), 1.1 g; Mn (manganese sulphate), 600 mg; I (as calcium iodate), 6 mg; Co (as cobalt chloride), 10 mg; Se (as sodium selenite), 2 mg.

### 2.2. Sample collection and feed composition

After the feeding trial, 5 lambs were selected randomly from each group and transported to a nearby abattoir for traditional halal slaughter. The rumen, jejunum, cecum, and colon were separated by ligation with cotton thread to prevent the contents from moving between adjoining sections, their contents were collected with sterile forceps into 2 mL sterile lyophilization tubes, stored in liquid nitrogen, and brought to the laboratory for storage at-80°C.

The chemical composition of GP and feeds was determined following the Association of Official Analytical Chemists (AOAC International, 2007), and included DM (934.01), ether extract (945.16), crude protein (990.03, Kjeldahl, N × 6.25), calcium (927.02), and phosphorus (965.17). The NDF (assayed with a heat-stable amylase) and ADF were measured using the filter bag technique with a fiber analyzer (A2000; ANKOM Technology, NY, USA), as described by Van Soest et al. (1991) (Table 2).

**Table 2:** Dry matter intake (DMI), body weights, average daily gain (ADG) and feed conversion ratio (FCR) in lambs consuming control (CD) and grape pomace (GP) diets.

Items	CD	GP	SEM^1^	*p*-Value
DMI, g/d	1527^b^	1580^a^	6.25	<0.001
Initial weight, kg	22.6	22.4	0.40	0.806
Final weight, kg	31.1	30.4	0.61	0.574
ADG, g/d	186	175	11.75	0.670
FCR	8.21	9.03	0.63	0.731

1 SEM, standard error of mean.

^a,b^Means within a row with different lowercase letters differ from each other (*p* < 0.05).

### 2.3. DNA extraction, amplification, and sequencing

The cetyltrimethylammonium bromide method was used to extract DNA from each sample, and subsequent determination of DNA purity and concentration used agarose gel electrophoresis. The polymerase chain reaction (PCR) was done with the V3-V4 region of bacteria and the V4-V5 region of archaea as targets. After PCR product mixing and purification, PCR products were extracted using the library constructed by the TruSeq® DNA PCR-Free Sample Preparation Kit, and after Qubit and real-time PCR quantification, the library was sequenced using NovaSeq6000.

### 2.4. Bioinformatics analysis

The QIIME 2 (Bolyen et al., 2019) was used for microbiome bioinformatics. The q2-demux plugin was used to demultiplex and quality filter the raw sequence data; the feature table was clustered by 97% similarity using Vsearch (Rognes et al., 2016). All the operational taxonomic units (OTUs) were aligned using the mafft (Katoh et al., 2002), and were used to construct a phylogenic tree using fasttree2 (Price et al., 2010). Taxonomy was assigned to OTUs using the q2-feature-classifier (Bokulich et al., 2018), classify-sklearn naïve Bayes taxonomy classifier against the Silva 138 99% reference sequences (McDonald et al., 2012) to classify and annotate OTUs. Filtering feature tables included total-frequency-based filtering by data volume, species-based filtering of tables and sequences. Alpha and beta diversities, microbial composition and MetaStat analysis of difference species calculation and visualization used the R package microeco (Liu et al., 2020); and functional prediction used phylogenetic investigation of communities by reconstruction of unobserved states (PICRUSt2) (Douglas et al., 2020) based on the metabolic pathways from all domains of life (MetaCyc) database (Caspi et al., 2020).

### 2.5. Statistical analyses

The ADG, DMI and FCR were compared between dietary groups using a one-way ANOVA (SPSS 26, Chicago, IL, USA). Alpha diversity indices were compared between groups using the Wilcox test, beta diversity was analyzed using multivariate analysis of variance (PERMANOVA), differences in the relative abundances of the rumen, jejunum cecum, and colonic microbiota at the genus level were tested by MetaStat analysis (Paulson et al., 2011), for different site differential pathway analysis of treatments used the Welch’s *t*-test test, correlations between differential microbiota and MetaCyc pathways used the “corr.test” function in R4.2.2, and false discovery rate (FDR)-corrected Spearman’s correlation test and visualized used the Pheatmap R package. A level of *p* < 0.05 was accepted as significant and 0.05 < *p* < 0.10 as tended to be significant.

## 3. Results

### 3.1. Effect of grape pomace on average daily gain, dry matter intake and feed conversion ratio in Tan lambs

The DMI was greater (*p* < 0.05) in the GP than CD group, but there was no difference (*p* > 0.05) between the two groups in initial and final body weights, ADG and FCR (Table 2).

### 3.2. Grape pomace altered gastrointestinal bacterial community structure

A total of 3,441,427 and 3,460,722 paired-end sequencing reads were obtained from four different parts of the GIT of the lambs using 16S rRNA gene sequencing for bacterial V3-V4 region and archaeal V4-V5 region, and 8,253 sequences and 6,430 OTUs were obtained by quality control and Vsearch clustering. In addition, 2055 and 297 OTUs were obtained by total-frequency-based filtering (samples with a total frequency of less than 31,488 and all features with a total abundance of less than ten were filtered), contingency-based filtering (filtered the feature present in at least two samples), and taxonomy-based filtering of tables (removed all features that contain either mitochondria or chloroplast, excluded the archaea at kingdom-level annotation in bacterial results and the bacteria in archaeal results) for subsequent analyses.

**Figure 1:**
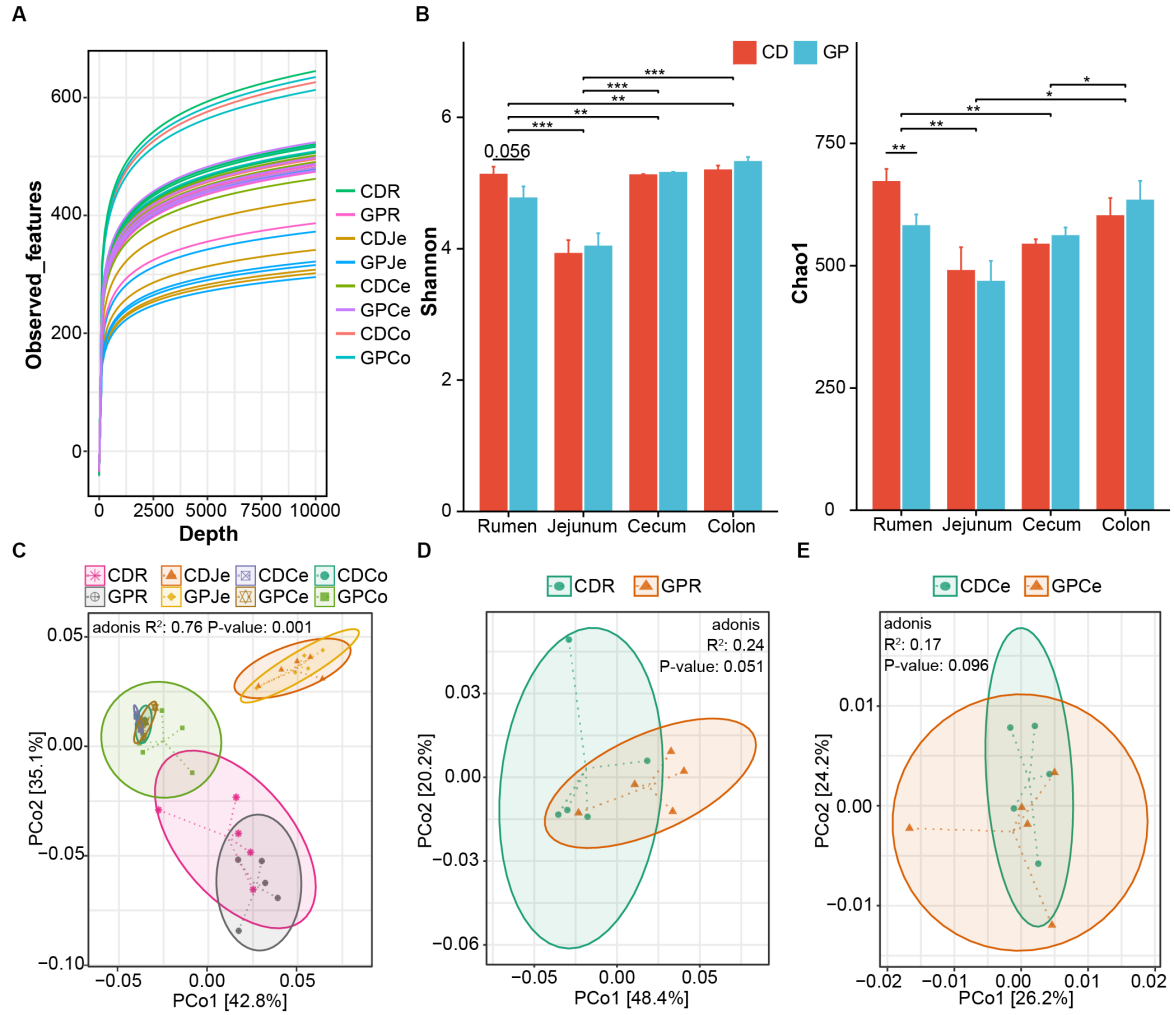
Effect of control (CD) and grape pomace (GP) diets on the bacterial community structure of the gastrointestinal tract (GIT). **(A)** The rarefaction curve calculated by observed features; **(B)** Alpha diversity representing species richness and evenness using Chao1 and Shannon indices of CD and GP in GIT; Principal-coordinate analysis (PCoA) of the GIT **(C)**, rumen **(D)** and cecum **(E)** bacteria community at OTUs level by PERMANOVA analysis based on weight uniFrac distances. CDR, control diets rumen; GPR, GP rumen; CDJe, control diets jejunum; GPJe, GP jejunum; CDCe, control diets cecum; GPCe, GP cecum; CDCo, control diets colon; GPCo, GP colon. Data are presented as mean ± standard error of mean (SEM) (*n* = 5). ***p < 0.001, **0.001 < *p* < 0.01, *0.01 < *p* < 0.05.

The first set of questions examined the feasibility of sequencing. The construction of the rarefaction curve (Figure 1A) demonstrated that the curve tended to plateau and, therefore, the sequencing data were large enough to reflect most of the microbial diversity information in the samples. Next, we analyzed the effect of diet and GIT position on alpha diversity (Figure 1B). The Chao1 index was lesser (*p* = 0.008) and the Shannon index tended to be lesser (*p* = 0.056) in the rumen of the GP than in CD lambs. In the GIT, the Shannon (*p* = 0.0005) and Chao1 indices (*p* = 0.031) in the rumen were greater than in the jejunum, and both were greater than in the cecum (Shannon: *p* = 0.031, Chao1: *p* = 0.006) and colon (Shannon: *p* = 0.006). The Shannon index of the jejunum was lesser than the cecum (Shannon: *p* < 0.001) and colon (Shannon: *p* < 0.001, Chao1: *p* = 0.046), and Chao1 index of the cecum was lesser than the colon (*p* = 0.046). We compared the beta diversity of microorganisms in CD and GP groups in different GIT sites; they were different (*p* = 0.001) and the figure separated the rumen, jejunum, and hindgut at the OTU level (Figure 1C). Only in the rumen (*p* = 0.051) and cecum (*p* = 0.096) did the bacterial composition tend to differ between the CD and GP groups (Figures 1D,E).

### 3.3. Composition and biomarkers of gastrointestinal bacteria in different treatments

**Figure 2:**
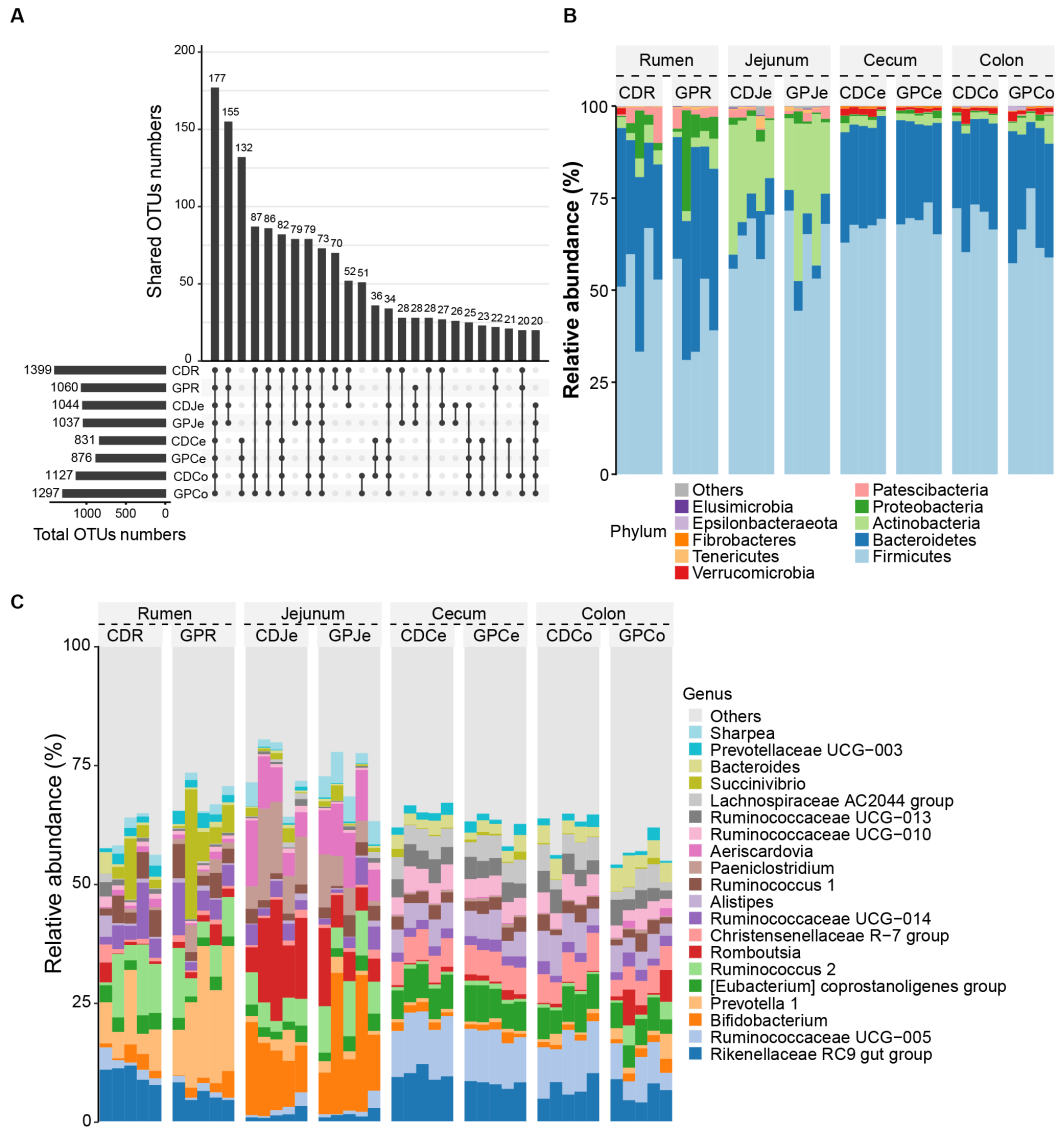
Composition of the gastrointestinal tract bacteria in each group. **(A)** The upset diagram in different treatments for different sites; **(B)** Top 10 bacteria at phylum level; **(C)** Top 20 bacteria at the genus level. CDR, control diet rumen; GPR, grape pomace diet rumen, CDJe, control diet jejunum; GPK, GP jejunum; CDCe, control diets cecum; GPCe, GP cecum; CDCo, control diets colon; GPCo, GP colon.

We analyzed the overlap and differences of the OTUs between the CD and GP groups. The number of OTUs in the GP group was lesser in the rumen and jejunum, but greater in the cecum and colon than in the CD group. Upset plots also display the zonal changes in bacterial OTUs at different sites and similarities in adjacent sites (Figure 2A). The dominant phyla (Figure 2B) were Firmicutes (CD: 52.7%, GP: 43.0%) and Bacteroidetes (CD: 35.2%, GP: 41.3%) in the rumen; Firmicutes (CD: 63.8%, GP: 60.4%), Actinobacteria (CD: 23.7%, GP: 29.4%) and Bacteroidetes (CD: 7.43%, GP: 6.22%) in the jejunum; Firmicutes (CD: 66.9%, GP: 69.1%) and Bacteroidetes (CD: 27.9%, GP: 26.4%) in the cecum; and Firmicutes (CD: 68.7, GP: 64.3%) and Bacteroidetes (CD: 26.6%, GP: 28.6%) in the colon. The dominant genera (Figure 2C) were *Prevotella 1* (CD: 8.57%, GP: 20.3%), *Rikenellaceae RC9 gut group* (CD: 10.0%, GP: 5.68%) in the rumen; *Bifidobacterium* (CD: 13.4%, GP:18.0%), *Romboutsia* (CD: 15.4%, GP:7.84%), *Paeniclostridium* (CD: 11.42%, GP: 5.76%) in the jejunum; *Ruminococcaceae UCG-005* (CD: 11.35%, GP:10.50%), *Rikenellaceae RC9 gut group* (CD: 9.92%, GP:7.88%) in the cecum; and *Ruminococcaceae UCG-005* (CD: 10.62%, GP:6.69%), *Rikenellaceae RC9 gut group* (CD: 6.97%, GP: 6.34%), in the colon.

**Figure 3:**
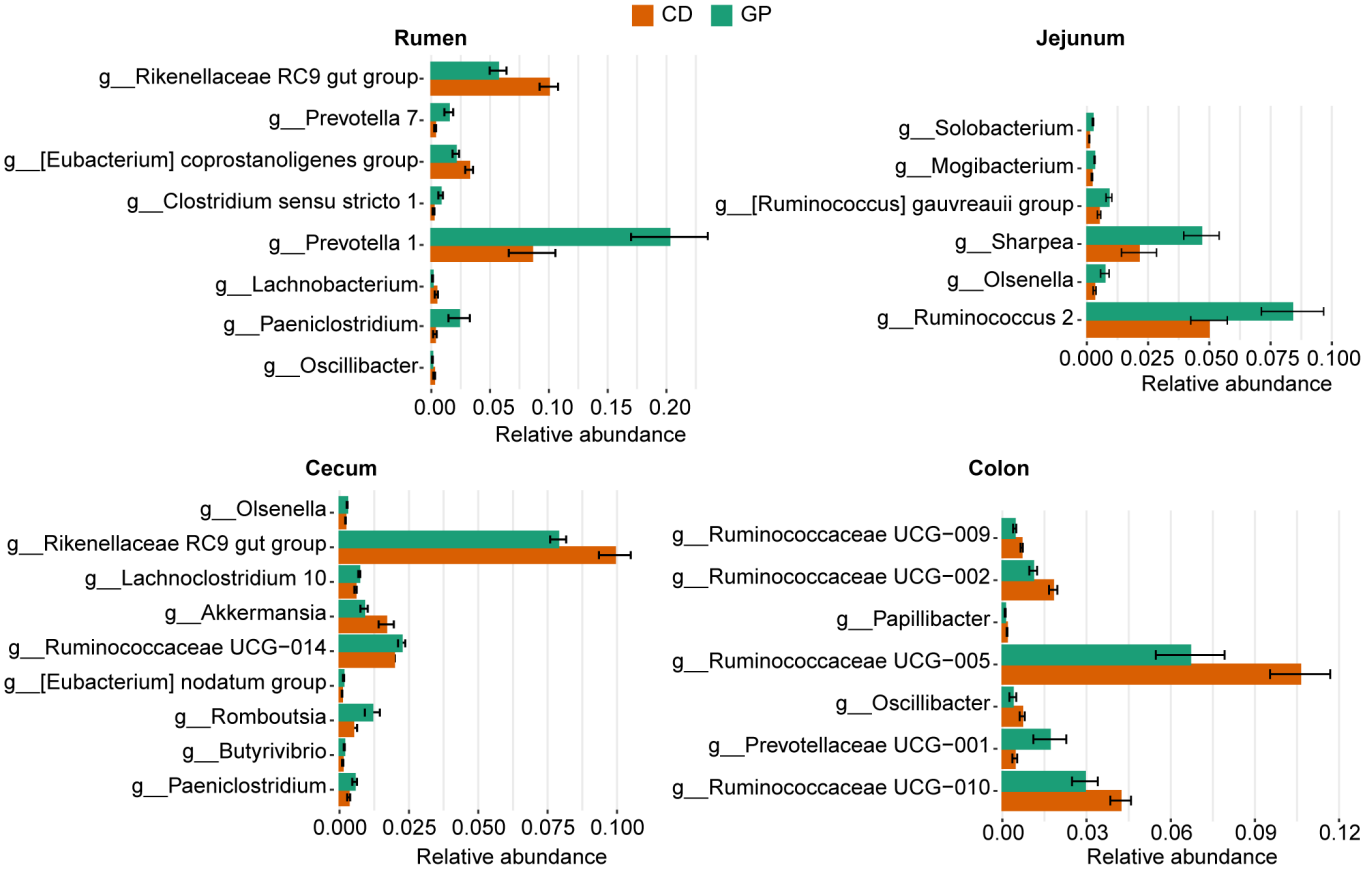
Bacteria at the genus level with significant differences in relative abundances (>0.1%) between control (CD) and grape pomace (GP) groups in the rumen, jejunum, cecum, and colon. Data are presented as mean ± standard deviation (SD) (*n* = 5). The bars are arranged in descending order of value of *p*.

We screened for biomarkers at the four sites of the GP group. MetaStat analysis (Figure 3) revealed that there were differences between GP and CD in the GIT. Compared to the CD group, the relative abundances of *Prevotella 1* (*p* = 0.01), *Prevotella 7* (*p* = 0.008), *Paeniclostridium* (*p* = 0.030), and *Clostridium sensu stricto 1* (*p* = 0.009) increased in the rumen of the GP group, while *Rikenellaceae RC9 gut group* (*p* = 0.02) and *[Eubacterium] coprostanoligenes group* (*p* = 0.008) decreased. *Prevotella 1* displayed the greatest variation in the rumen. The abundance of several bacteria in the jejunum increased in the GP group, including *Ruminococcus 2* (*p* = 0.041), *Sharpea* (*p* = 0.023), *[Ruminococcus] gauvreauii group* (*p* = 0.016), *Olsenella* (*p* = 0.037) and *Mogibacterium* (*p* = 0.008). The abundance of *Ruminococcaceae UCG-014* (*p* = 0.030), *Romboutsia* (*p* = 0.039), *Lachnoclostridium 10* (*p* = 0.020) and *Paeniclostridium* (*p* = 0.046) were greater in the cecum, and the abundance of *Rikenellaceae RC9 gut group* (*p* = 0.018), *Akkermansia* (*p* = 0.023) were lesser in the GP than CD group. In the colon of the GP group, similar trends were exhibited in that the abundance of *Ruminococcaceae UCG-005* (*p* = 0.028), *Ruminococcaceae UCG-010* (*p* = 0.049), *Ruminococcaceae UCG-009* (*p* = 0.006), *Ruminococcaceae UCG-002* (*p* = 0.006) and *Oscillibacter* (*p* = 0.043) were lesser, but of *Prevotellaceae UCG-001* (*p* = 0.049) was greater than in the CD group.

### 3.4. PICRUSt2 predicted significant differences in Metacyc pathways

The functions in gastrointestinal bacteria following GP feed were predicted using PICRUSt2, in which the sequences and abundances were input, and gene family and pathway abundances were output. The functional predictions based on the MetaCyc database of metabolic pathways and enzymes obtained 388 pathways.

**Figure 4:**
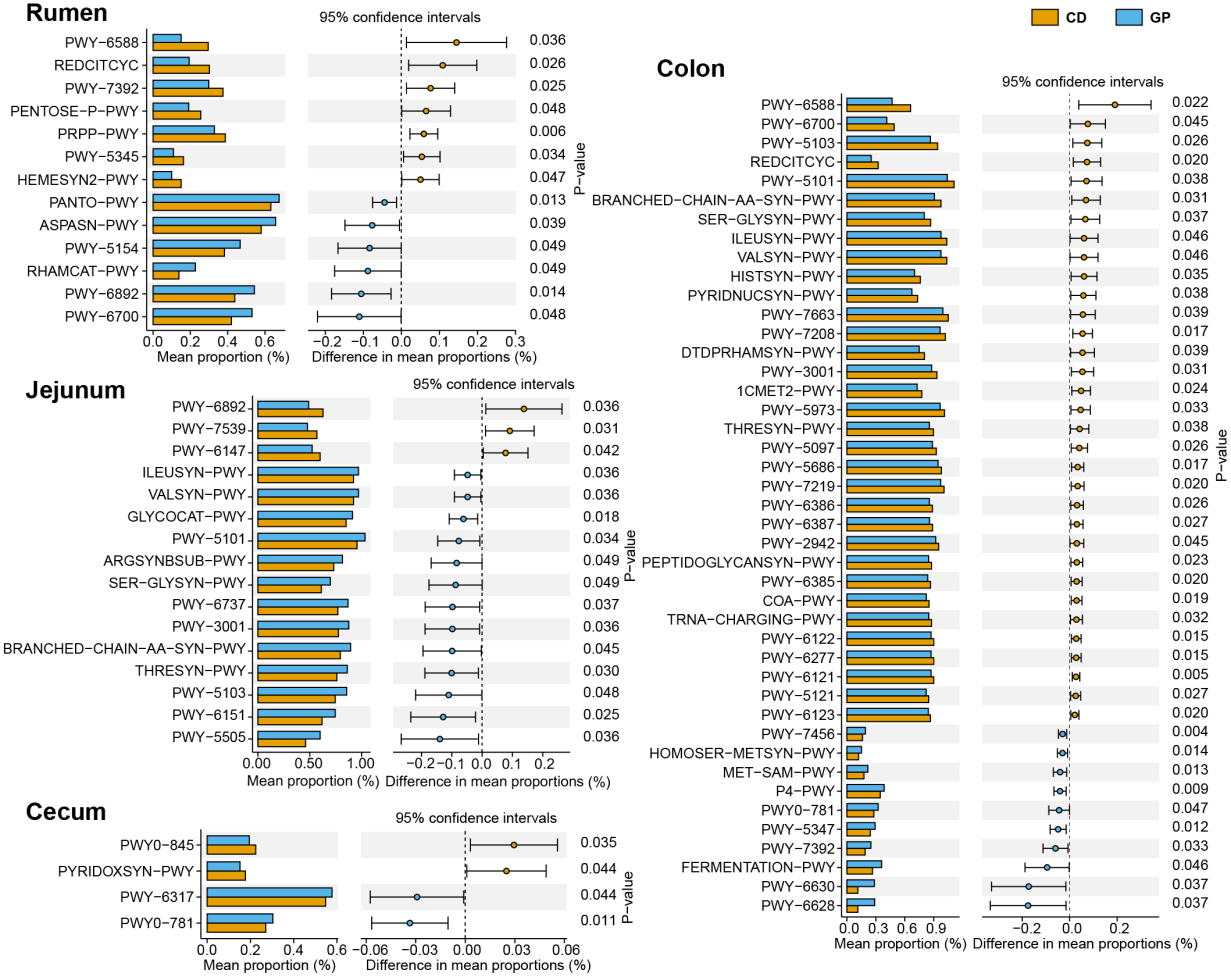
Significantly different MetaCyc pathways in rumen, jejunum, cecum, and colon. The error bars on the columns represent the standard error of mean (SEM).

The differential pathways (>0.1%) after treatment were analyzed using Welch’s t-test in each region (Figure 4), with the most differential pathways in the colon and least in the cecum. In the rumen, the GP up-regulated the ASPASN-PWY (superpathway of L-aspartate and L-asparagine biosynthesis), PWY − 5,154 (L-arginine biosynthesis III) involved in vitamin biosynthesis, amino acid biosynthesis: PANTO−PWY (phosphopantothenate biosynthesis I), PWY − 6,892 (thiazole biosynthesis I) and carbohydrate degradation: and RHAMCAT−PWY (L-rhamnose degradation I); and down-regulated sugar metabolism PWY − 6,588 (pyruvate fermentation to acetone), REDCITCYC (TCA cycle VIII), PENTOSE−P − PWY (pentose phosphate pathway), PRPP−PWY (superpathway of histidine, purine, and pyrimidine biosynthesis), and PWY − 5,345 (superpathway of L-methionine biosynthesis). In the jejunum, the GP group up-graded the pathways in polysaccharide degradation: GLYCOCAT-PWY (glycogen degradation I), PWY-6737 (starch degradation V); but down-graded 11 pathways of amino acid metabolism: branched amino acid biosynthesis (ILEUSYN-PWY, ILEUSYN-PWY, PWY-5103, VALSYN-PWY, BRANCHED-CHAIN-AA-SYN-PWY), arginine (ARGSYNBSUB-PWY), serine (SER-GLYSYN-PWY) e.g., folate biosynthesis (PWY-7539, PWY-6147) and thiamine biosynthesis (PWY-6892). In the cecum, the pathways of PWY-6317 (galactose degradation I), PWY0-781 (aspartate superpathway) were up-graded, but vitamin biosynthesis (PYRIDOXSYN-PWY, PWY0-845) was down-regulated. The GP up-regulated seven amino acid metabolism pathways (HOMOSER-METSYN-PWY, P4-PWY, PWY0-781, PWY-5347, PWY-6630, PWY-6628), mannan degradation (PWY-7456), and mixed acid fermentation (TFERMENTATION-PWY); and down-regulated 11 pathways linked with amino acid metabolism such as L-isoleucine biosynthesis (PWY-5101, ILEUSYN-PWY, PWY-5103), L-valine biosynthesis (VALSYN-PWY), L-lysine biosynthesis (PWY-2942); carbohydrate metabolism: TCA cycle VIII (REDCITCYC), nicotinamide adenine dinucleotide biosynthesis I (PYRIDNUCSYN-PWY), pyruvate fermentation to acetone (PWY-6588); cofactor, carrier, and vitamin biosynthesis (COA-PWY, 1CMET2-PWY, PYRIDNUCSYN-PWY) and nucleoside and nucleotide biosynthesis (PWY-5686, PWY-6122, PWY-6277, PWY-7208, PWY-7219) in the colon.

### 3.5. Correlation of bacteria at the genus level and MetaCyc pathways

**Figure 5:**
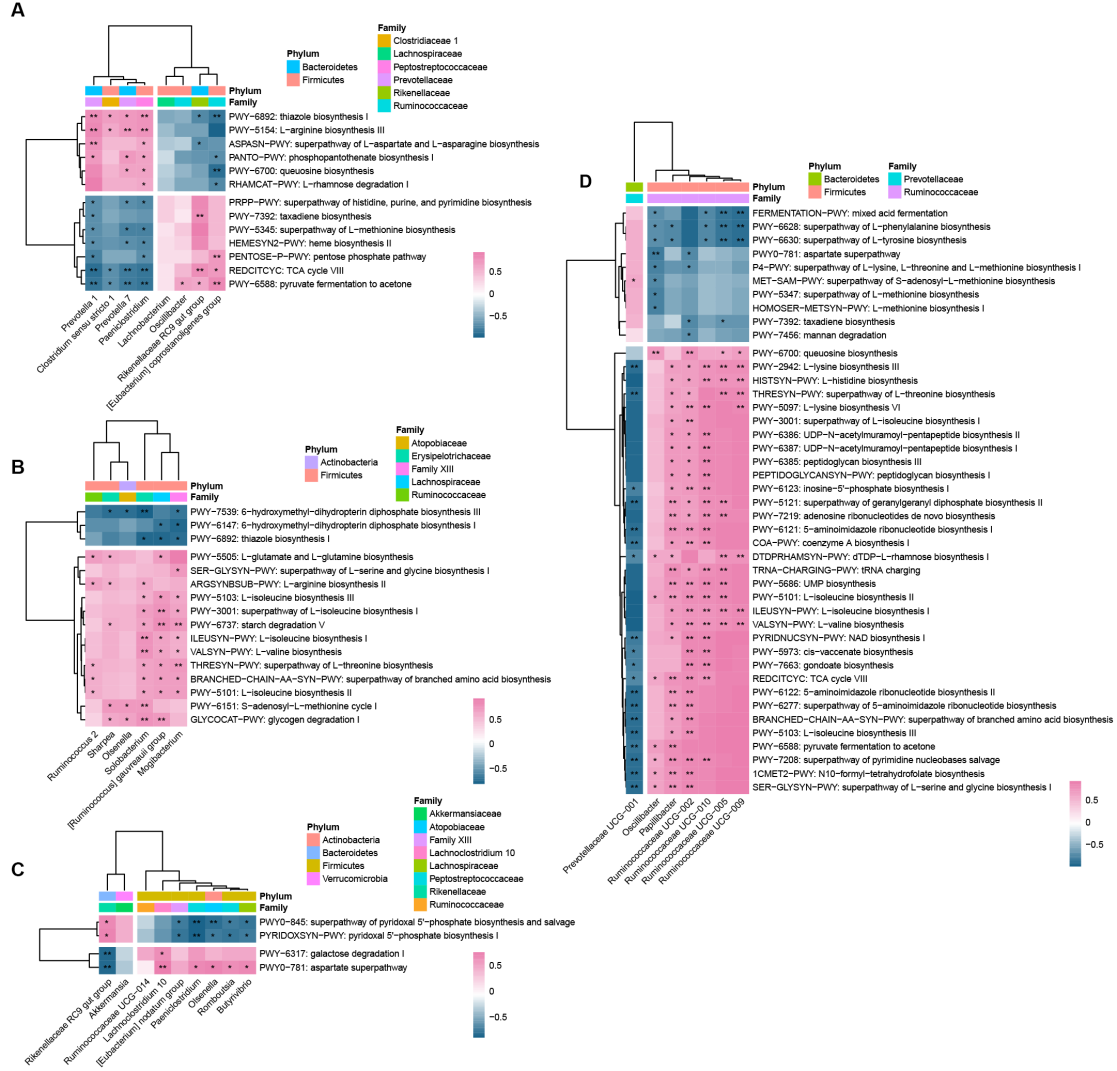
Spearman correlations between genera and pathways in rumen **(A)**, jejunum **(B)**, cecum **(C)**, and colon **(D)**. For each heatmap, rows correspond to bacteria at the genus level, columns correspond to MetaCyc pathways. Red and blue represent the positive and negative correlations, respectively. The intensity of the colors denotes the degree of correlation between the genera abundances and the pathways. **p < 0.01, * *p* < 0.05.

There were strong correlations between most of the bacteria and pathways in different sites (Figure 5). In general, there was a positive correlation between up-graded pathways and the genera that were most abundant in CD; whereas, the less abundant genera in CD displayed negative correlations. In general, there were positive correlations between up-graded pathways and the genera that were most abundant in CD, while, the least abundant genera in CD were correlated negatively. For rumen bacteria, the GP-altered microbes exhibited a functional distinction. In GP lambs, the relative abundances of the rumen *Prevotella 1*, *Prevotella 7*, *Paeniclostridium*, and *Clostridium sensu stricto 1* were correlated positively with the up-regulated pathways of L-arginine biosynthesis, queuosine biosynthesis, peptidases, and inhibitors, glycerophospholipid metabolism, and phosphopantothenate biosynthesis, and negatively with the down-regulated pathways. There were positive correlations between the low abundant bacteria in CD, including the *Rikenellaceae RC9 gut group*, *[Eubacterium]coprostanoligenes group*, and *Oscillibacter* with pyruvate fermentation to acetone, TCA cycle Vlll, and the down-regulated pathways. However, there were negative correlations between these bacteria and the up-regulated pathways. In the jejunum, the six significantly different bacteria species were all greater in the GP than CD group. In the jejunum, the GP group had six bacteria species with greater abundance than in the CD group; those genera were correlated positively with amino acid biosynthesis, starch and glycogen digestion. *Solobacterium*, *[Ruminococcus] gauvreauii group* and *Mogibacterium* were correlated positively with most up-regulated pathways. The main changed microorganisms of jejunum were correlated negatively with folate biosynthesis (PWY-7539, PWY-6147) and thiamine biosynthesis (PWY-6892). In the cecum, the *Rikenellaceae RC9 gut group* was correlated positively with vitamin biosynthesis (PYRIDOXSYN-PWY, PWY0-845), but negatively with the up-graded pathways (galactose degradation and aspartate superpathway). *Romboutsia*, *Olsenella*, *Paeniclostridium*, *Butyrivibrio* were correlated positively with vitamin biosynthesis (PYRIDOXSYN-PWY, PWY0-845), and negatively with the down-graded pathways. In the colon, the genus *Prevotellaceae UCG-001* was correlated positively with MET-SAM-PWY (superpathway of S-adenosyl-L-methionine biosynthesis), *Prevotellaceae UCG-001* was correlated negatively with the down-graded pathways, while *Ruminococcaceae UCG-002*, *Ruminococcaceae UCG-010*, and *Papillibacter* were correlated positively with most down-regulated pathways. *Ruminococcaceae UCG-005* and *Ruminococcaceae UCG-09* were correlated positively with isoleucine (ILEUSYN-PWY, PWY-5101), valine (VALSYN-PWY), lysine (PWY-2942, PWY-5097), histidine (HISTSYN-PWY), and threonine (HRESYN-PWY) biosynthesis.

### 3.6. The difference in gastrointestinal archaea diversity and biomarkers

Alpha and beta diversities of archaea were used to characterize species diversity within and between GIT segments. Sequencing analysis of archaea from different segments of the GIT revealed that the Shannon index (*p* = 0.056) tended to be greater in the rumen of GP than CD (Supplementary Figure S1A); however, the Chao1 index did not differ between dietary treatments in the different sites of the GIT. The beta diversity of each site (Supplementary Figure S1B) also displayed differences (*p* = 0.001), with the GP diets altering the beta diversity of the jejunum (*p* = 0.006) and colon (*p* = 0.008) at the OTUs level (Supplementary Figures S1C,D).

To characterize the archaeal composition, the relative abundances of the top 10 phyla and genera were charted. At the phylum level (Supplementary Figure S2A), in the rumen and jejunum, Euryarchaeota (CDR:95.4%, GPR:91.1%, CDJe:99.1%, GPJe:97.2%) dominated, followed by Halobacterota (CDR:0.16%, GPR: 5.05%, CDJe:0.69%, GPJe:2.47%) and Thermoplasmatota (CDR:4.56%, GPR: 3.32%, CDJe:0.23%, GPJe:0.34%), while in the cecum and colon, Halobacterota (CDCe:46.8%, GPCe:42.5%, CDCo:16.1%, GPCo:56.5%) and Euryarchaeota (CDCe:53.0%, GPCe:56.8%, CDCo:83.8%, GPCo:42.9%) dominated. At the genus level (Supplementary Figure S2B), *Methanobrevibacter* (CDR:95.2%, GPR: 91.3, CDJe:99.7%, GPJe:97.2%) dominated in the rumen and jejunum, followed by *Methanocorpusculum* (CDR:0.10%, GPR:4.80%, CDJe:0.67%, GPJe:2.46%) and *Methanosphaera* (CDR:0.90%, GPR:2.60%, CDJe:4.20%, GPJe:6.17%); and in the cecum and colon, *Methanocorpusculum* (CDCe:95.2%, GPCe:91.3, CDCo:99.7%, GPCo: 97.2%) and *Methanobrevibacter* (CDCe:52.4%, GPCe:56.0%, CDCo:83.3%, GPCo: 42.0%) dominated, followed by *Methanocorpusculum* (CDCe:46.8%, GPCe:42.5%, CDCo:15.9%, GPCo:52.3%).

The Metastats analysis detected the change of archaea in different sites in the GIT at the genus level (Supplementary Figure S2C). The dominant genera were *Methanobrevibacter* and *Methanocorpusculum*, but the change in the cecum was from other genera. *Methanobrevibacter* was lesser in the rumen (*p* = 0.01), jejunum (*p* = 0.06) and colon of GP (*p* = 0.007), but greater in the cecum (*p* = 0.007) in the CD than GP group. Moreover, the abundance of *Methanocorpusculum* was greater in the rumen (*p* = 0.03), jejunum (*p* = 0.06) and colon (*p* = 0.009) and tended to be lesser in the cecum (*p* = 0.09) in the GP than CD group.

**Figure 6:**
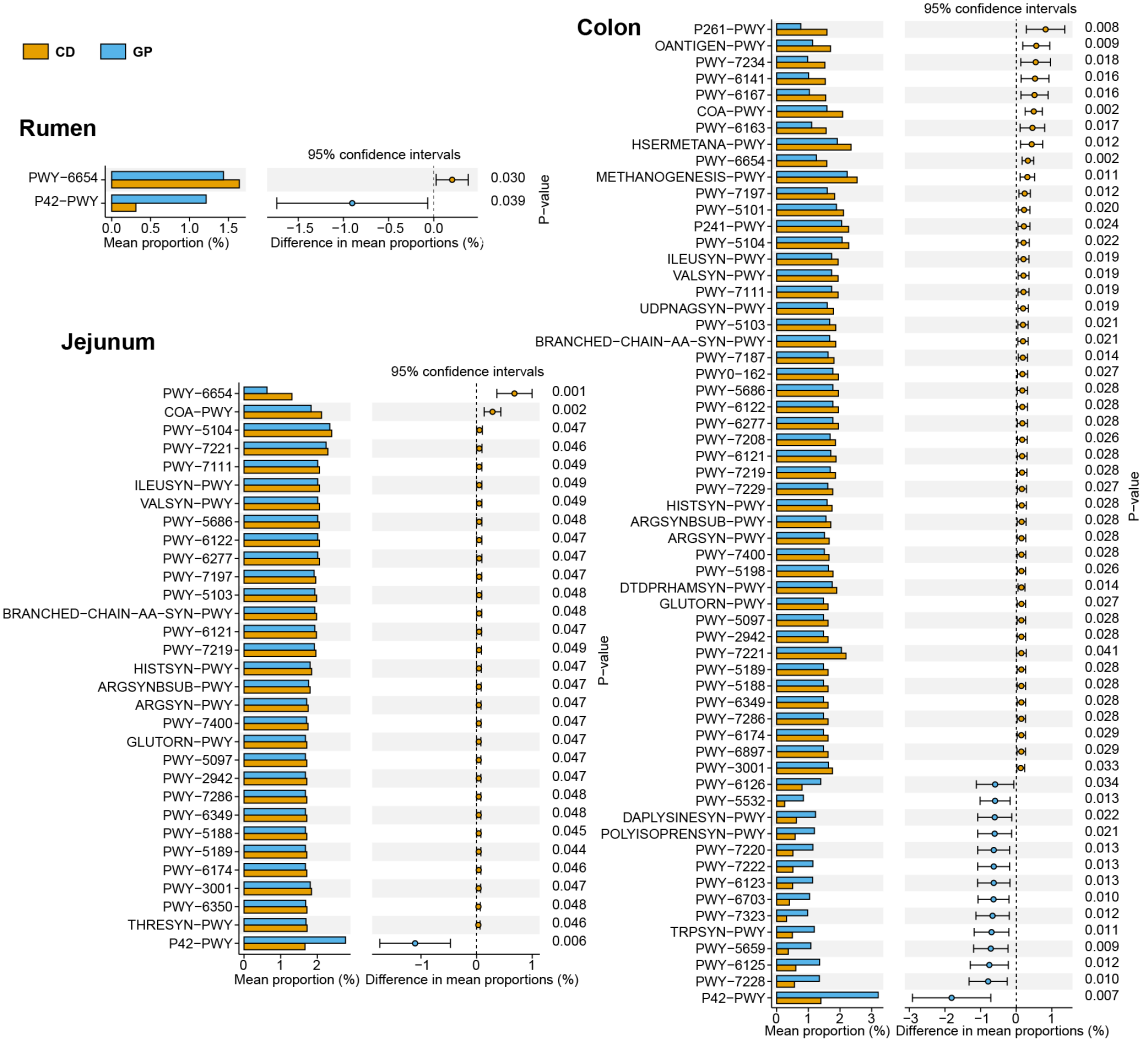
Different archaeal prediction pathways at different regions in Tan lambs after consuming grape pomace. The error bars on the columns represent the standard error of mean (SEM).

We used PICRUSt2 to predict changes in archaeal function in different GIT sites by the MetaCyc database, and 262 pathways were obtained. The relative abundances of pathways greater than 0.1% were tested for differences between dietary groups at each GIT site. The pathways of the jejunum and colon were distinct, whereas the cecum did not show any difference (Figure 6). The incomplete reductive TCA cycle (P42-PWY) was up-regulated, while phosphopantothenate biosynthesis III (PWY-6654) was down-regulated in the rumen of the GP group. In the jejunum in the GP group, only the pathway of incomplete reductive TCA cycle (P42-PWY) was up-regulated, while 14 amino acid metabolism pathways, including arginine biosynthesis (PWY-7400, ARGSYN-PWY, ARGSYNBSUB-PWY), isoleucine biosynthesis (PWY-5104, ILEUSYN-PWY, PWY-5103, PWY-3001), and lysine biosynthesis (PWY-2942, PWY-5097), were down-regulated. In the colon, the GP group had a greater enrichment of pathways in the carbohydrate metabolism as incomplete reductive TCA cycle (P42-PWY), guanosine diphosphate mannose biosynthesis (PWY-5659), superpathway of guanosine diphosphate mannose-derived O-antigen building blocks biosynthesis (PWY-7323), and 5 purine nucleotide biosynthesis, including superpathway of guanosine nucleotides *de novo* biosynthesis I (PWY-7228), superpathway of guanosine nucleotides *de novo* biosynthesis II (PWY-6125), adenosine deoxyribonucleotides *de novo* biosynthesis II (PWY-7220), guanosine deoxyribonucleotides *de novo* biosynthesis II (PWY-7222), and superpathway of adenosine nucleotides *de novo* biosynthesis II (PWY-6126). Furthermore, methanogenesis from H_2_ and CO_2_ (methanogenesis-PWY), 15 amino acid biosynthesis such as lysine (PWY-2942, PWY-5097), arginine (PWY-7400, ARGSYN-PWY, ARGSYNBSUB-PWY), methionine (HSERMETANA-PWY), isoleucine (PWY-3001, PWY-5103, ILEUSYN-PWY, PWY-5104, PWY-5101), six co-factor, carrier, and vitamin biosynthesis: factor 420 biosynthesis (PWY-5198), co-enzyme M biosynthesis I (P261-PWY), co-enzyme B biosynthesis (P241-PWY), thiamin salvage II (PWY-6897), flavin biosynthesis II (PWY-6167), co-enzyme A biosynthesis I (COA-PWY); and pyruvate fermentation to isobutanol (PWY-7111) were down-regulated.

## 4. Discussion

Dietary composition is the main factor influencing gastrointestinal microbiology. In the present study, GP reduced the alpha diversity and altered the community structure of rumen bacteria, and the community structure of jejunal and colonic archaea. Host metabolism was affected by altering the relative abundances of some bacteria (*Prevotella*, *Rikenellaceae RC9 gut group*, and *Ruminococcus*) and archaea (*Methanobrevibacter* and *Methanocorpusculum*). The GP induced alterations in the bacteria associated with fermentation in the rumen and colon and changes in their metabolic functions and fermentation patterns, while inhibiting the metabolic activity of colonic archaea.

Under iso-energetic and iso-nitrogenous conditions, replacing 8% of corn with GP and increasing soybean oil in the diet resulted in higher DMI. The high energy soybean oil (16.2 g at 34.7 kJ DE/g) was added to compensate for the low energy GP (80 g at 6.8 kJ DE/g), so that the diets would be iso-energetic and iso-nitrogenous. This increase in intake in the GP diet could be attributed to the potential improvement in palatability due to the soybean oil (Macleod and Buchanan-Smith, 1972). Similar to a previous study, a dietary GP of approximately 10% resulted in an increased DMI in sheep (Zhao et al., 2018). Comparison of the two iso-energetic and iso-nitrogenous diets, a diet with 8% GP did not improve growth performance or FCR, and, consequently our first hypothesis was not supported. However, the present results are consistent with other studies that reported diets with less than 20% GP did not affect FCR in sheep (e.g., Chikwanha et al., 2019). The FCR in the Tan lambs in the present study was relatively high, 8.21 and 9.03; but similar results have been reported in other studies. For example, Liu et al. (2021) reported a FCR of 9.1 with *ad libitum* intake and a range between 8.1 and 10.3 with different intakes for Tan sheep. Tan sheep are hardy and well adapted to a dry, cold and windy environment, but FCR is poor.

Broadly consistent with previous studies (Zhang et al., 2018; Xie et al., 2021), bacteria and archaea of the GIT displayed spatial heterogeneity in community structure and composition, as he GIT microbiome differed among rumen, small intestine, and hindgut. The alpha diversity of rumen bacteria decreased, while there were marked changes in the bacterial community structure with the consumption of GP. These results differed from those of Rolinec et al. (2023) who reported that the consumption of a diet with 1 and 2% GP increased alpha diversity among rumen bacteria and stimulated higher levels of methanogen abundance. The difference among studies may be attributed to differing levels of dietary GP. It is possible that the polyphenols in GP inhibit bacteria. Polyphenols have a stronger bactericidal effect on gram-positive bacteria than on gram-negative bacteria due to the structure of their cell walls and the arrangement of their outer membranes. These polyphenols modify the membrane’s permeability and integrity of the cell walls of gram-negative bacteria in the rumen (Vasta et al., 2019).

In the present study, although the dominant bacterial species in the rumen and colon differed, their bacteria and functions were similar. *Prevotella 1* was the dominant bacterial genus in the rumen of sheep (Henderson et al., 2015; Li et al., 2020). This genus degrades carbohydrates (starch, hemicellulose, and pectin) and proteins to produce succinic and acetic acids (Krieg et al., 2010; Accetto and Avguštin, 2019). The association of *Prevotella* with propionate production has been reported previously (Strobel, 1992; Purushe et al., 2010; Betancur-Murillo et al., 2023). Propionate is the major substrate for hepatic gluconeogenesis in ruminants, with an energy utilization efficiency of 109% (Orskov, 2012), competes for hydrogen in propionate production by *Prevotella*, and also reduces the production of methane (Denman et al., 2015). *Ruminococcaceae* are the dominant fiber fermentation bacteria in the GIT of sheep, producing mainly acetate (Peng et al., 2021; Ma et al., 2022). The reduction in fiber-degrading bacteria may be due to the binding of tannins to the fibers or their inhibitory effect on microbial activity. Tannins can prevent microorganisms from attaching to the fiber, and gram-positive specialized fibrolytic bacteria are very susceptible to tannins (McSweeney et al., 2001). Previous research demonstrated that distiller’s dried grains with solubles (DDGS) and tannin-rich diets reduced the abundance of rumen *Ruminoccoccaceae* and increased the abundance of *Prevotella* (Khiaosa-ard et al., 2015; Pannell et al., 2022), as was observed in the present study.

The up-regulated pathways in the rumen and colon were all related to glycolysis. L-rhamnose, a part of complex pectin polysaccharides, produced pyruvate upon degradation and increased propionate production in *in vivo* and *in vitro* studies (Hosseini et al., 2011). Mannan, a major component of plant hemicellulose (Moreira and Filho, 2008), promotes the glycolytic pathway, that is, the mixed acid (from glycolysis) fermentation to lactate, succinate, and acetate. The plant structural carbohydrates and hemicellulose are rich in pentose, and down-regulating the pentose phosphate pathway decreases acetate. Down-regulation of the TCA cycle inhibits acetyl-co-enzyme A conversion to reduce further the precursors for acetate and butyrate synthesis (Ungerfeld, 2020). Furthermore, thiamin diphosphate and phosphopantothenate, also known as vitamin B1 and B5, were up-regulated pathways, and, therefore, GP promotes the synthesis of B-vitamins in the rumen. The microbial synthesis of vitamin B in the rumen meets the vitamin requirement of the host and is a co-factor for various enzymes involved in the metabolism of fatty acids, carbohydrates, and amino acids. Thiamin diphosphate is an essential co-factor for a variety of enzymes, such as transketolase, pyruvate dehydrogenase, pyruvate decarboxylase, and α-ketoglutarate dehydrogenase (Lawhorn et al., 2004). Phosphopantothenate is an essential precursor for the synthesis of co-enzyme A, a co-factor in many intermediate metabolic pathways (Begley et al., 2001).

The jejunum digests and metabolizes starch and absorbs nutrients. In the GP group, the dominant genera belonged mainly to Firmicutes, which up-regulated bacterial starch, glycogen degradation, and amino acid biosynthesis. *Sharpea* and *[Ruminococcus] gauvreauii group* enhance the degradation of starch and glycogen. *Sharpea* ferments starch and melibiose to produce lactic acid and CO_2_ (Morita et al., 2008); lactic acid reduces gut pH and inhibits the proliferation of pathogenic bacteria, and, consequently, *Sharpea* is beneficial for the host’s health (Xu et al., 2018; Ma et al., 2020). *Ruminococcus 2* (family Ruminoccoccaceae) ferments starch to produce propionate (Tong et al., 2018; Chen et al., 2021), while *[Ruminococcus] gauvreauii group*, (Lachnospiraceae) ferments glucose into acetic acid (Domingo et al., 2008). *Mogibacterium* ferments starch and produces phenylacetate, but does not ferment carbohydrates (Schleifer, 2009), and its abundance increased when fed a high grain diet (Liu et al., 2015). The up-regulation of several amino acid synthesis pathways in the jejunum may be related to the polyphenols in GP, which bind to proteins, slowing down the degradation in the rumen and increasing protein metabolism in the small intestine (Guerra-Rivas et al., 2017).

The ruminant hindgut, which includes the cecum and colon, also ferments carbohydrates. However, differences in cecal bacteria and archaea between CD and GP were minor. The abundance of *Rikenellaceae RC9 gut group* decreased, but of *Ruminococcaceae UCG-014*, *Romboutsia* and *Lachnoclostridium* increased in the GP group. GP enriched the galactose degradation and aspartate superpathway. It was reported that the abundance of *Ruminococcaceae UCG-014* and *Olsenella* were correlated positively with feed efficiency in the rumen (Elolimy et al., 2020; McLoughlin et al., 2020). There was an increased abundance of these two bacteria in the cecum in the GP lambs, which may have promoted carbohydrate fermentation and improved feed efficiency. *Olsenella* was the only Actinobacterium with substantial variation, increased in both jejunum and cecum, and was correlated positively with glycogen and galactose depletion. *Olsenella* ferments starch and glycogen substrates to produce lactic, acetic, formic, and succinic acids (Göker et al., 2010; Kraatz et al., 2011). *Paeniclostridium* increased in both the rumen and cecum, and correlated with altered pathways in the rumen. Although this bacterium is potentially pro-inflammatory, as it produces hemorrhagic toxins that cause loss of gastrointestinal tissue and body disease (Lee et al., 2020), it was reported to be the predominant bacterium in the rumen of sheep (Zhao et al., 2023). *Paeniclostridium* has not been found in *in vivo* studies in which GP or polyphenols was fed to sheep. Some potential roles of *Paeniclostridium* were discovered in the bovine intestine, where the glycolytic pathways were up-regulated by the increased abundance of *Paeniclostridium* and *Romboutsia* (Sim et al., 2022). There appears to be a relationship betwee*n Paeniclostridium*, *Romboutsia*, or *Clostridium sensu stricto 1* and the fermentation process as they are the dominant bacteria in avocado oil processing waste feedstocks and dark fermentative hydrogen production (Yang and Wang, 2019; Ijoma et al., 2022). *Clostridium sensu stricto 1* can be fermented to produce butyric acid (Wang et al., 2017).

*Rikenellaceae RC9 gut group* and *[Eubacterium] coprostanoligenes group* are the main bacteria that decreased in relative abundances with GP. *Rikenellaceae RC9 gut group* is associated with rumen cellulose degradation, and it was reported that the ruminal abundance decreased in sheep fed high grain diets and diets with low NDF (Zened et al., 2013; Zhang et al., 2017). The *[Eubacterium] coprostanoligenes group* reduces cholesterol to coprostanol and ferments carbohydrates to produce acetic, formic and succinic acids (Freier et al., 1994). The ruminal abundance increased in animals fed a high energy, high lignin diet (Khatoon et al., 2022), and these bacteria were correlated positively with the TCA cycle, pentose phosphate pathway, and pyruvate fermentation to acetone. GP reduced the abundance of the *Rikenellaceae RC9 gut group* and the *[Eubacterium] coprostanoligenes group*, which may affect carbohydrate metabolism. We, therefore, propose that GP increases the abundance of propionate-producing and starch-fermenting bacteria and pathways, and decreases cellulose metabolism, thereby altering gastrointestinal fermentation, which supports our second hypothesis. Previous studies demonstrated that GP promotes propionate production and reduces acetate concentration (Foiklang et al., 2016a,b). The cashew nut shell liquid is rich in polyphenols, as is grape pomace. It has a comparable effect on rumen fermentation and reduces methane emissions (via hydrogenotrophic methanogenesis) by directly inhibiting hydrogenotrophic methanogenesis and reducing the copy number of the mcrA gene, fiber degradation, and the resulting H_2_ production (Shinkai et al., 2012).

Archaea are the only methane producers in the GIT (Janssen and Kirs, 2008). Four main types of methanogenic pathways have been identified: the pathway from H_2_ and CO_2_, the aceticlastic pathway from acetate, methanogenesis from methylated compounds, and methanogenesis from methoxylated aromatic compounds. The H_2_ and CO_2_ and methyltropic pathways are the main sources of soluble methane production in the gut (Beauchemin et al., 2020). The rumen and hindgut are the main sites of methane production, where methanogens play an essential role. In the GIT (except cecum), the abundance of *Methanobrevibacter* decreased, of *Methanocorpusculum* increased, and the pathways of incomplete reductive TCA cycle were up-regulated. The differences in archaea and pathways between CD and GP were particularly noticeable in the colon. Consistent with findings of earlier studies (Henderson et al., 2015), the most common genus of archaea was *Methanobrevibacter*, which can convert H_2_ or acetate to produce methane (Leahy et al., 2013). *Methanocorpusculum* occurs in the hindgut and feces of cattle and sheep and is associated with methane production (Ngetich et al., 2022), but the mechanism of action warrants further study. GP can inhibit methane production, due to the polyphenols, such as tannins, which inhibit methanogens and protozoa, and reduce fiber degradation and H_2_ production (Moate et al., 2014). In the current study, GP decreased the relative abundance and function of *Methanobrevibacter* in the GIT, which would support our third hypothesis. Methanogens are incapable of a complete oxidative or reductive TCA cycle, and incomplete cycles produce biosynthetic intermediates (Goodchild et al., 2004). However, in response to anaerobic or microaerophilic growth conditions, these incomplete cycles can still convert pyruvate into necessary biosynthetic intermediates (Wood et al., 2004). In the colon, the pathways of methanogenesis from H_2_ and CO_2_, the essential co-factors (co-enzyme M, co-enzyme B, and factor 420) of methanogenesis were down-regulated. All catabolic processes in methanogenesis require the reduction of the methylated form of co-enzyme M to methane by co-enzyme B, and factor 420 also acts as an electron carrier (Deppenmeier, 2002). GP lowers methane production by increasing propionate-producing microbes and pathways.

## 5. Conclusion

The current study demonstrated that a diet containing 8% dietary GP by dry weight increased DMI but did not affect ADG and FCR in Tan lambs. The GP enhanced the abundance of *Prevotella 1*, *Ruminococcus 2* and *Sharpea*, decreased the acetate-producing *Ruminococcaceae* and methane-producing *Methanobrevibacter*, stimulated starch metabolism, mixed acid fermentation, and intestinal amino acid and rumen B-vitamins biosynthesis, and down-regulated colonic methanogenesis. Overall, GIT microbiome altered fermentation to promote propionate production and degradation of starch, decrease cellulose fermentation and methanogenesis. In spite of these positive effects on the energy balance of the lambs, the ADG and FCR were not improved with a dietary supplement of 8% GP. Future studies with different GP levels are warranted. It is possible that levels of less than 8% GP would be beneficial for the entire GIT, reduce overall methane production and improve lamb performance.

## Data availability statement

The datasets presented in this study can be found in online repositories. The names of the repository/repositories and accession number(s) can be found below: https://www.ncbi.nlm.nih.gov/, PRJNA877028.

## Ethics statement

The animal studies were approved by the Academic Committee of the Northwestern Institute of Eco-Environment Resources, Chinese Academy of Sciences. The studies were conducted in accordance with the local legislation and institutional requirements. Written informed consent was obtained from the owners for the participation of their animals in this study.

## Author contributions

XC: Investigation, Software, Writing — original draft, Visualization. XD: Investigation, Formal analysis, Writing — review & editing. YL: Investigation, Formal analysis, Writing — review & editing. AD: Writing — review & editing. XW: Investigation, Software, Writing — review & editing. KJ: Investigation, Software, Writing — review & editing. QG: Methodology, Resources, Writing — review & editing. GX: Methodology, Resources, Writing — review & editing. HC: Methodology, Resources, Writing — review & editing. GY: Conceptualization, Resources, Writing — review & editing, Project administration and Funding acquisition.
