# Supplementary material for: Effect of grape pomace supplement on growth performance, gastrointestinal microbiota, and methane production in Tan lambs
Source: Front Microbiol. 2023 Sep 28;14:1264840. doi: 10.3389/fmicb.2023.1264840 (PMC10569316; doi:10.3389/fmicb.2023.1264840)
Supplement: Supplementary file 1 [file Data_Sheet_1.pdf]

Supplementary Material

1 Supplementary Figures

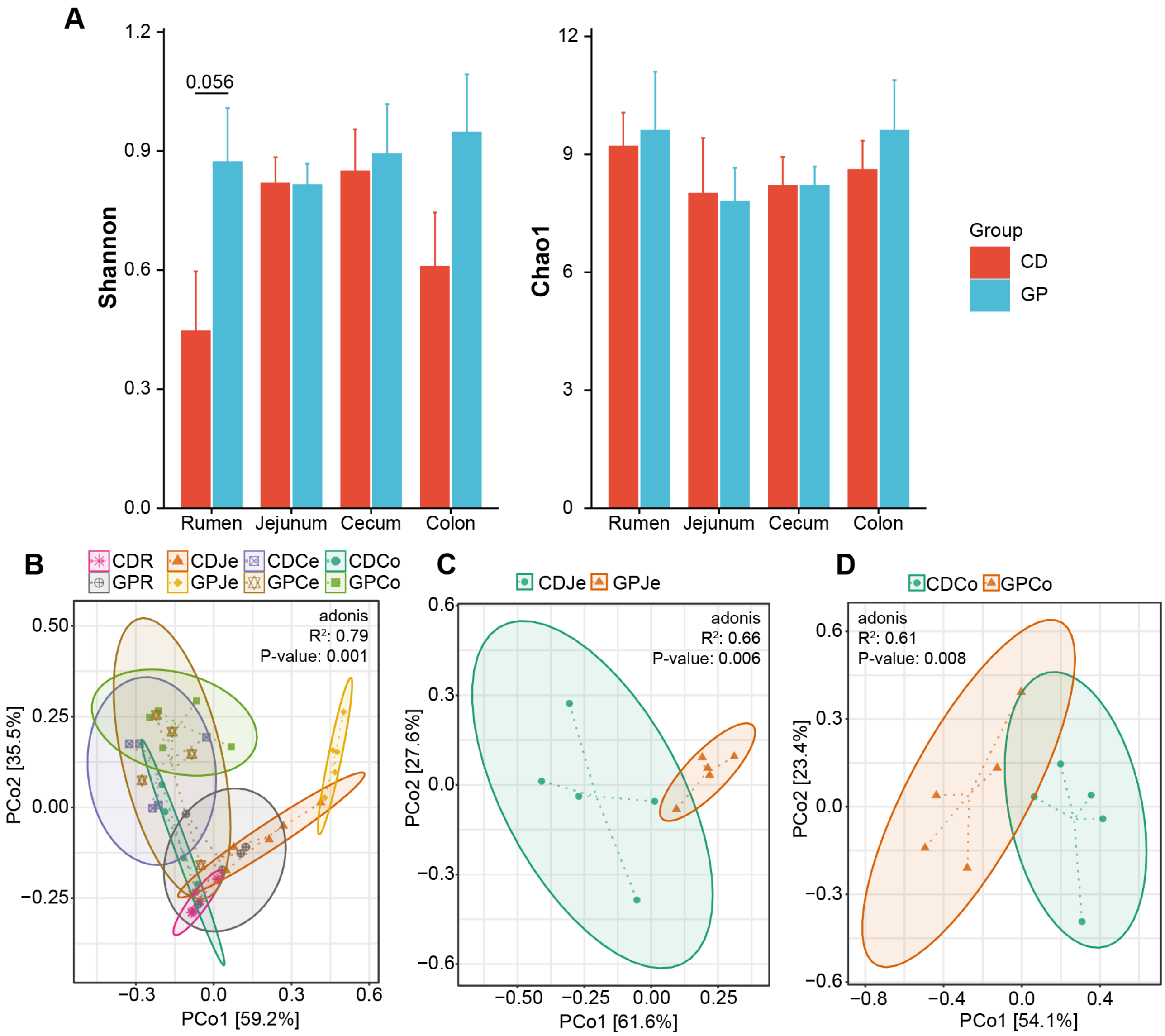

Supplementary Figure 1. Effect of grape pomace (GP) diet on the gastrointestinal tract (GIT) archaea composition. (A) Shannon index and Chao1 index of control diet (CD) and GP (grape pomace) group in GIT archaea; (B) PCoA of the GIT archaeal community in each group; (C) PCoA in the jejunum; (D) PCoA in the colon; PCoA based on weight UniFrac distances by PERMANOVA test. CDR: control diets rumen, GPR: GP rumen, CDJe: control diets jejunum, GPJe: GP jejunum, CDCe: control diets cecum, GPCe: GP cecum, CDCo: control diets colon, GPCo: GP colon. Data presented as means± standard error of mean (SEM). * $P<0.05$, ** $P<0.01$.

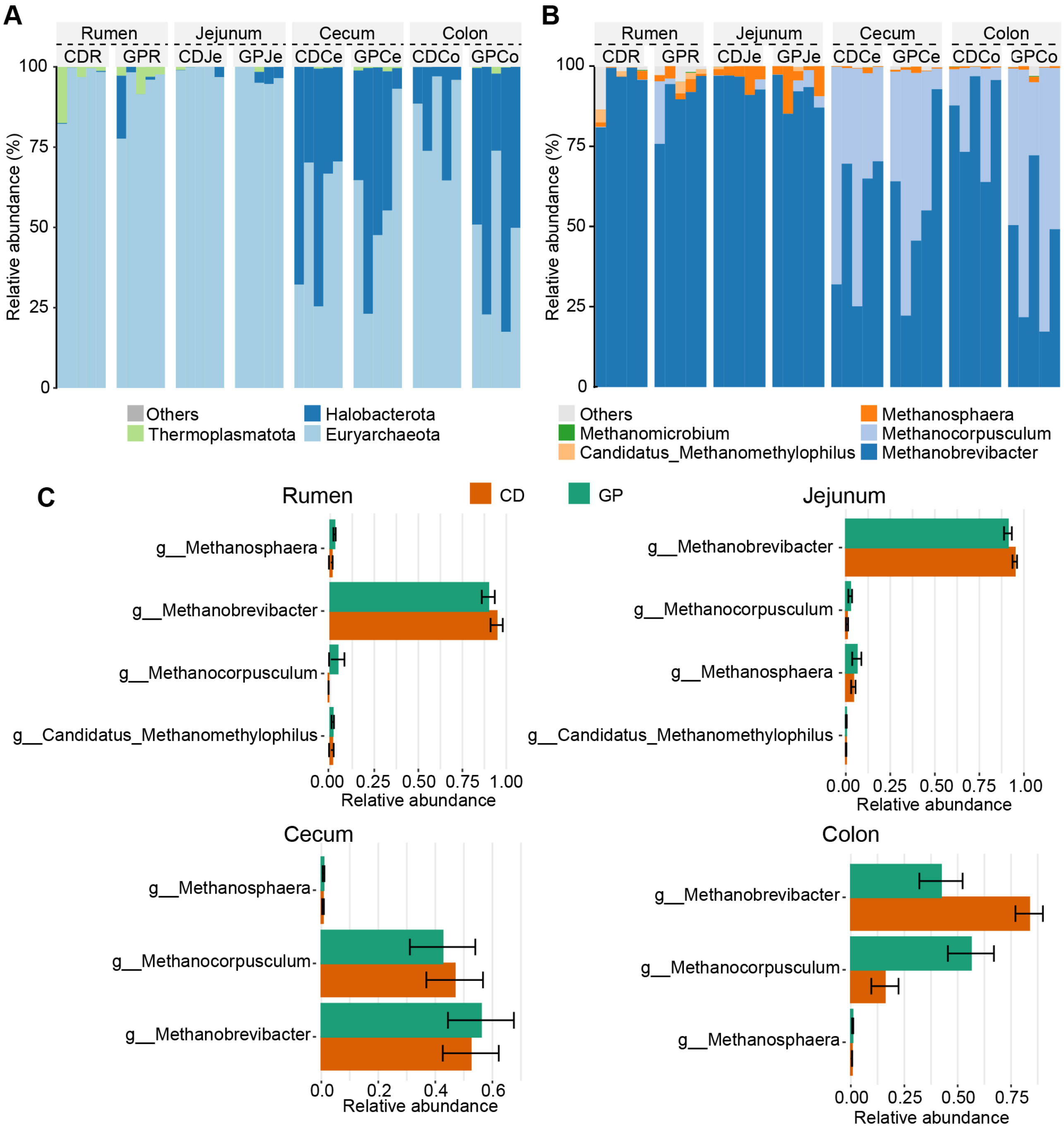

Supplementary Figure 2. Composition and biomarkers of archaea between control (CD) and grape pomace (GP) diets. (A) TOP 10 archaea at phylum level; (B) TOP 10 archaea at genus level; (C) Significantly differential abundances between CD and GP group at the archaeal genus level. Data presented as means± standard deviation (SD). CDR: control diets rumen, GPR: GP rumen, CDJe: control diets jejunum, GPJe: GP jejunum, CDCe: control diets cecum, GPCe: GP cecum, CDCo: control diets colon, GPCo: GP colon.
